# Response of Mouse Zygotes Treated with Mild Hydrogen Peroxide as a Model to Reveal Novel Mechanisms of Oxidative Stress-Induced Injury in Early Embryos

**DOI:** 10.1155/2016/1521428

**Published:** 2016-09-22

**Authors:** Diting Qian, Zhiling Li, Yuting Zhang, Yue Huang, Que Wu, Gaizhen Ru, Man Chen, Bin Wang

**Affiliations:** Reproductive Center, The First Affiliated Hospital of Shantou University Medical College, Shantou University, Shantou, Guangdong, China

## Abstract

Our study aimed to develop embryo models to evaluate the impact of oxidative stress on embryo development. Mouse zygotes, which stayed at G1 phase, were treated with prepared culture medium (containing 0.00, 0.01, 0.02, 0.03, 0.04, 0.05, or 0.1 mM hydrogen peroxide (H_2_O_2_)) for 30 min in experiment 1. The dose-effects of H_2_O_2_ on embryo development were investigated via comparisons of the formation rate at each stage (2- and 4-cell embryos and blastocysts). Experiment 2 was carried out to compare behaviors of embryos in a mild oxidative-stressed status (0.03 mM H_2_O_2_) with those in a control (0 mM H_2_O_2_). Reactive oxygen species (ROS) levels, variation of mitochondrial membrane potential (MMP), expression of *γ*H2AX, and cell apoptosis rate of blastocyst were detected. We observed a dose-dependent decrease on cleavage and blastocyst rates. Besides, higher level of ROS, rapid reduction of MMP, and the appearance of *γ*H2AX revealed that embryos are injured early in mild oxidative stress. Additionally, *γ*H2AX may involve during DNA damage response in early embryos. And the apoptotic rate of blastocyst may significantly increase when DNA damage repair is inadequate. Most importantly, our research provides embryo models to study cell cycle regulation and DNA damage response under condition of different levels of oxidative stress.

## 1. Introduction

In aerobic respiration cell, ROS, such as H_2_O_2_, hydroxyl radical (^•^OH) and superoxide anion radical (O_2_^−^), appears as a by-products of cell metabolism [1]. Physiological concentration of ROS is necessary to maintain normal embryo development [2, 3]. However, excessive ROS, produced in improper culture conditions, such as culture systems [4], light conditions [5], and gas atmosphere [1, 6–8], may cause developmental arrest, DNA damage, and cell apoptosis [1]. In many species studied, evidences have been found that ROS compromises embryo development [4–8]. In contrast with gassed bags, incubator chamber is more efficient in generating higher number and better quality of bovine embryos due to a lower level of ROS [4]. Hamster embryos will stop dividing at two-cell stage when zygotes are exposed to cool white fluorescent light [5]. Besides, the number of cleaved embryos is similar when embryos are cultured under 5% or 20% O_2_ [7, 8]. But there is a decline in the percentage of blastocyst formation [7, 8]. We can learn that human embryos on day 3 are apparently normal in both groups, but embryo quality is prior in 5% O_2_ [8]. Those studies revealed that ROS-induced damage correlates with the phenomenon above.

ROS displays roles of double-edged sword in many physiological and pathological processes in cell [9]. It affects embryo development by altering gene expression [1], inducing lipid peroxidation and DNA damage [1, 9], containing nuclear and mitochondrial DNA strand breaks [9, 10]. Numerous studies used H_2_O_2_ to simulate deleterious effects of ROS on embryo development. H_2_O_2_, a form of highly active ROS, readily penetrates the cellular envelope and forms ^•^OH which is a more active form of ROS. Liu et al. chose an intensive (1 mM H_2_O_2_ for 1.5 h) and a mild (0.2 mM for 15 min) stimulus to treat zygotes (fertilized in vivo); embryos from both groups do not divide till 96 hpi [11]. In another experiment, 2-cell embryos were treated with different doses of H_2_O_2_ (0–50 *μ*M) for 30 or 60 min; the results showed a negative dose-effect on blastocyst rate [12]. We may observe a decline in blastocyst rate when treating zygotes with 35 *μ*M H_2_O_2_ for 15 min [13]. Researches on embryos injured by ROS are common, but most of their embryos was collected from in vivo fertilization, and/or part of the oxidative stimulus started at a later stage of embryo development. Those are inconsistent with clinical assisted reproductive techniques which eggs fertilize in vitro and embryos suffer oxidative stress from the very first.

Studies state that accumulation of ROS caused developmental arrest of embryos by altering activities and structures of mitochondria [10, 11, 14]. Firstly, alteration of mitochondria is an early event in the process of oxidative damage [11]. Secondly, mild oxidative stress may induce a decline in MMP and dysfunction of mitochondria. Then cell cycle arrest and programmed cell death follow [10, 11, 15]. Lastly, mitochondria play an important role in mediating embryo development and apoptosis in oxidative status [10]. Thus, we regard decrease of MMP as an indicator of mitochondrial damage and the early phase of apoptosis.

Another important inducement of embryo developmental retardation is DNA damage. Our previous studies found that *γ*H2AX, the marker of DNA damage [16–18], appeared at 1- and 4-cell embryos fertilized with H_2_O_2_-treated sperm [19]. Further experiments revealed that oxidative stress in sperm activated G2/M delay by cascade of ATM → Chk1 → Cdc25B/Cdc25C to repair damaged DNA [19–21]. It has been identified that damage response checkpoints include G1/S, intra-S, and G2/M [22]. Cell cycle delay gains time for DNA repair. Besides, *γ*H2AX provides other molecules such as ATM, Rad 50, and Rad 51 with a recognition site to activate DNA damage response [16–18, 23]. Those molecules are key to repair DNA damage. But specific molecular mechanisms of developmental delay in embryos with oxidative-stressed damage are obscure. Also, cell cycle checkpoint and apoptosis share some common molecules [24]. To some extent, cells will end with death if DNA damage does not repair [25].

The phenomenon above pushes us to research the molecular mechanisms of developmental retardation in zygotes with oxidative-stressed damage. Because of the limitation of clinical researches on human embryos, we treated mouse zygotes with different doses of exogenous H_2_O_2_ to simulate effects of oxidative stress on embryonic development. In order to clarify the molecular mechanisms of DNA damage response activated by oxidative stress and cell cycle arrest in early embryos in subsequent experiment, we aim to develop alternative embryo models with oxidative damage in this research.

## 2. Materials and Methods

### 2.1. Animals

Adult Kun-Ming mice (3–6 weeks old) were purchased from the Animal Center of Shantou University Medical College and treated in compliance with the Guide for the Care of Use of Laboratory Animal by the US National Institutes of Health (NIH Publication number 85-23, revised 1996) and the rules of the National Animal Protection of China. All experimental protocols were approved by the Laboratory Animal Ethics Committee of our institution (SUMC2014-014). This study was approved by the Institutional Animal Care and Use Committee of Shantou University Medical College.

### 2.2. Reagents and Media

All reagents were purchased from Sigma (St. Louis, MO, USA) unless otherwise stated. Rabbit antibody for anti-phospho-histone H2AX (*γ*H2AX; Ser 139) and goat anti-rabbit Alexa Fluor 488 secondary antibody were from Abcam (England). The In Situ Cell Death Detection kit (Fluorescein) was from Roche (Switzerland). PI and DAPI were from Panera (France). Human tubal fluid-HEPES (HTF-HEPES) was from Cooper Surgical (USA). Phosphate-buffered saline (PBS) was dissolved in Milli-Q water: 136.9 mM NaCl, 2.7 mM KCl, 0.9 mM CaCl_2_, 0.5 mM MgCl_2_, 7 mM Na_2_HPO_4_, 1.25 mM NaH_2_PO_4_, 1.5 mM KH_2_PO_4_, and 1 g/L PVA (290 mOsm/kg; pH 7.2). Pancreatin solution was diluted to 0.1% with PBS, the pH adjusted to 3.0 with HCl, filtered, aliquoted, and stored at 4°C. The embryo culture medium was made by adding 0.4% BSA and 10% fetal bovine serum to HTF solution. And the medium of the treated group was made by addition of H_2_O_2_ to a final concentration of 0.01, 0.02, 0.03, 0.04, 0.05, or 0.1 mM and then preequilibrated them for 1 hour before use. Stationary liquid was 4% paraformaldehyde in PBS. Membrane liquid was PBS + 0.5% Triton X-100. Sperm capacitation liquid (HTF solution + 1.5% bovine serum albumin), fertilization liquid (HTF solution + 0.4% BSA), and embryo culture medium were placed at 37°C in a 5% CO_2_ incubator for 4 h. Blastocysts were cultured in blastocyst medium (Cooper Surgical, Inc.).

### 2.3. Epididymal Sperm Preparation, Collection, and Culture of Oocytes and Embryos

As described in our previous study, sperm was obtained from the tail of the epididymis of mature male mice and incubated in capacitation medium at 37°C in a 5% CO_2_ incubator for 1 h [19]. Female mice were intraperitoneally injected with consecutive injections of 10 IU pregnant mare serum gonadotropin (PMSG) to promote ovulation. And 10 IU human chorionic gonadotropin (HCG) 48 h apart. Then mice were euthanized by cervical dislocation at 13 to 15 h after HCG administration. Cumulus oocytes were collected from the ovaries of the mice, placed in 37°C PBS, then moved to 37°C fertilization liquid containing 10 *μ*L capacitated sperm, and incubated for 6 h to permit fertilization. Zygotes were washed three times and cultured in new medium after fertilization.

### 2.4. Experiment  1: Effects of Zygotes Oxidative Stress on Embryonic Development

According to studies by Liu et al. [11] and our preliminary experiments, zygotes (7 hpi) were placed in culture medium with different concentrations of H_2_O_2_ for 30 minutes, then rinsed three times, and cultured in medium without H_2_O_2_. Embryos were incubated at 37°C in a 5% CO_2_ atmosphere. The medium was renewed daily. Development of two-cell and four-cell embryos and blastocysts was observed, respectively, at 24 hpi, 48 hpi, and 96 hpi with an inverted microscope (Olympus Inc., Japan). Cleavage and blastocyst formation rates were defined as the total number of embryos from corresponding stage divided by total number of zygotes. The total number of zygotes included in 0.01, 0.02, 0.03, 0.04, 0.05, and 0.1 mM H_2_O_2_ was 519, 363, 316, 489, 365, 326, and 50, respectively.

### 2.5. Experiment  2: Effects of Oxidative Stress Induction on Embryo Injured Related Variables

#### 2.5.1. Determination of ROS Products

The intracellular ROS level was detected by 2′, 7′-dichlorofluorescein fluorescence (DCFH-DA). A stock solution of DCFH-DA (1 × 10^−3^ mol/L in DMSO) was added to the embryo culture medium to a final concentration of 10 *μ*mol/L, and zygotes were incubated at 37°C in 5% CO_2_ for 30 minutes. Embryos were rinsed three times (5 min each time) in fresh culture fluid to remove the residual dye and then placed on a glass slide with 20 *μ*L culture fluid. Fluorescence staining was observed immediately under a fluorescence microscope with exciting light of 495 nm and emissive light of 520 nm (Nikon Eclipse 90 Ni-E). The fluorescence intensity was recorded at 5 s after exciting the zygotes. Image Pro Plus 6.0 was used to quantify the fluorescence (Media Cybernetics Inc., Bethesda, MD, USA). 34 zygotes from the treated group and 29 zygotes from the untreated group were detected in this part.

#### 2.5.2. Detection of Mitochondrial Membrane Potential (MMP)

MMP was detected at 1-cell embryos with the lipophilic, cationic probe 5, 5′, 6, 6′-tetrachloro-1, 1′ 3, 3′-tetraethylbenzimidazolylcarbocyanine iodide (JC-1, stock 5 mg/mL). Then, PBS was added to the solution of JC-1 to a final concentration of 1.25 *μ*mol/L. Zygotes were washed three times with PBS (5 min each time) after staining for 20 min and placed on a glass slide. Images were recorded immediately by using a fluorescent microscope (Nikon Eclipse 90 Ni-E) at an excitation wavelength of 488 nm and beam path control setting at LP 585 nm for Ch1 and BP 505–530 nm for Ch2. Analysis of data was done with Image Pro Plus 6.0 software (Media Cybernetics Inc., Bethesda, MD, USA). The number of treated and untreated zygotes at each hour was shown as follows: 18 and 20 for 0 h, 17 and 25 for 1 h, 22 and 19 for 2 h, 22 and 21 for 4 h, and 19 and 16 for 6 h.

#### 2.5.3. Assay of *γ*H2AX

Embryos were rinsed with TPBS (PBS + 0.05% Tween 20), and zona pellucidae were removed from embryos with pancreatin (0.1%) for 30 s. Then embryos were fixed with 4% paraformaldehyde in TPBS for 30 min, permeabilized at room temperature for 30 min, and blocked for 1 h at room temperature in blocking solution (PBS + 0.05% Tween 20 + 3% BSA + 10% normal goat serum). Before each step, embryos were washed with TPBS three times, 5 minutes per time. After blocking, embryos were incubated with rabbit anti-*γ*H2AX primary antibody (1 : 1000 dilution) for 12 h to 15 h at 4°C, then washed, and incubated with goat anti-rabbit Alexa Fluor 488 secondary antibody (1 : 500 dilution). The immunostained embryos were washed with TPBS, counterstained with propidium iodide, and observed under a fluorescence microscope (Nikon Eclipse 90 Ni-E).

#### 2.5.4. TUNEL Assay

TUNEL assay was performed to analyze apoptosis of blastocysts, using the In Situ Cell Death Detection kit (fluorescein) in accordance with manufacturer's instructions. Embryos were withdrawn at 96 hpi to collect blastocysts according to our previous experiment [20]. Zona pellucidae were removed from the embryos at first. Then embryos were washed three times with TPBS, fixed in 4% paraformaldehyde at room temperature for 30 min, and mounted on polylysine slides and washed again with TPBS and permeabilized at room temperature for 30 min. Embryos were washed with TPBS three times again and incubated with fluorescein-conjugated dUTP and terminal deoxynucleotidyl transferase at 37°C in dark for 1 h. The reaction was terminated by washing in TPBS three times, 5 min each time, and then the embryos were counterstained with DAPI, rinsed with TPBS, and sealed with coverslips. Observation was done under an Olympus FluoView FV 1000 confocal microscope (Olympus Inc., Japan). According to previous studies [20], the apoptotic rate was expressed as the percentage of TUNEL-positive cell number relative to the total cell number of blastocysts. 24 blastocysts from the control group and 34 from the treated group were used for the TUNEL assay.

### 2.6. Statistical Analysis

Results were collected from at least 3 independent experiments and data were analyzed by SPSS 17.0 software (SPSS Inc., Chicago, IL). Data showed as percentages were analyzed using Chi square test. Values expressed as mean ± SD of treated and untreated groups was compared, using Student's *t*-test. *P* < 0.05 was considered statistically significant.

## 3. Results

### 3.1. Experiment  1: A Dose-Dependent Decline of Embryonic Development at Each Stage

**Figure 1:**
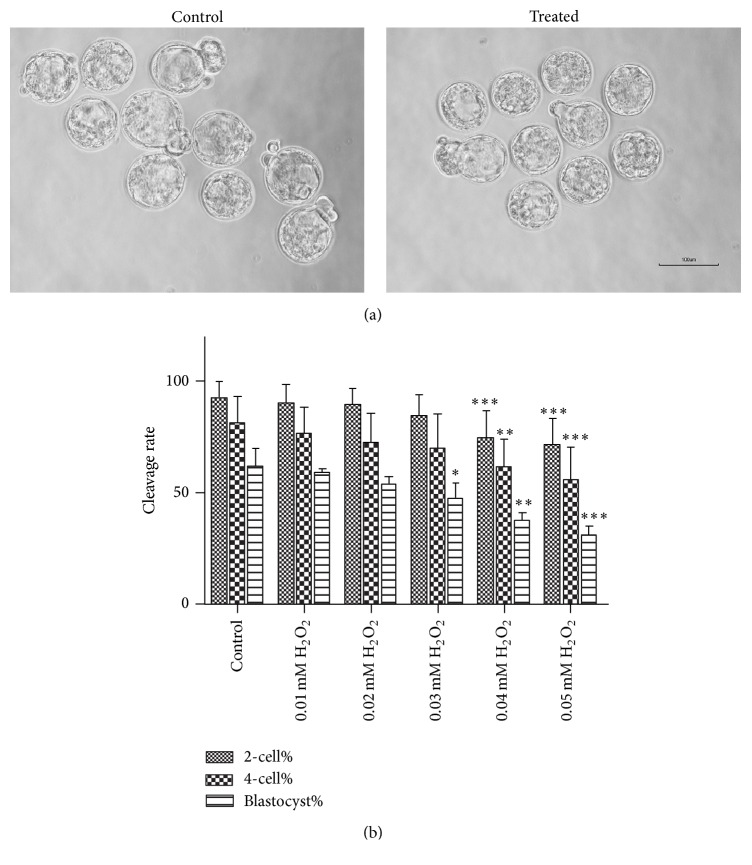
Comparisons of embryo development between zygotes from groups with different concentration of H_2_O_2_ and control group. (a) Representative images of embryos at 96 hpi from control group and 0.03 mM H_2_O_2_ group. (b) Counting out the cleavage rates from each group, blastocyst formation rate declined from 0.03 mM H_2_O_2_ group; early embryos showed stagnation from 0.04 mM H_2_O_2_ group. Data are presented as mean ± SD in six independent experiments for each group at least. Differences between the groups were calculated using Chi square test. ^*∗*^ *P* < 0.05; ^*∗∗*^ *P* < 0.01; ^*∗∗∗*^ *P* < 0.001 compared with the control group.

We could learn from Figure 1(a) that blastocysts have a bigger shape in control group than those in 0.03 mM H_2_O_2_ group. And we characterized the dose-response of H_2_O_2_ on embryo development. No zygotes divided at 60 hpi when they were treated with 0.1 mM H_2_O_2_. H_2_O_2_ at 0.01 mM or 0.02 mM had no significant effects on the percentages of two- and four-cell embryos or blastocyst formation at 24, 48, and 96 hpi, respectively (Figure 1(b)) while H_2_O_2_ at 0.03 mM exposure produced a 14.50% reduction in blastocyst formation rate (*P* < 0.05, Figure 1(b)). Although the two- and four-cell embryo formation rates were not different from the control group, 0.03 mM H_2_O_2_ was the concentration of our treated group in subsequent experiments. When mouse zygotes were exposed to H_2_O_2_ at 0.04 mM or 0.05 mM, the proportion of blastocyst formation and cleavage rates decreased (*P* < 0.05, Figure 1(b)) and exhibited a dose-dependent decline (Figure 1(b)).

### 3.2. Experiment  2: Oxidative Stress Implies the Variations in Embryos

#### 3.2.1. ROS Concentrations in Embryos

**Figure 2:**
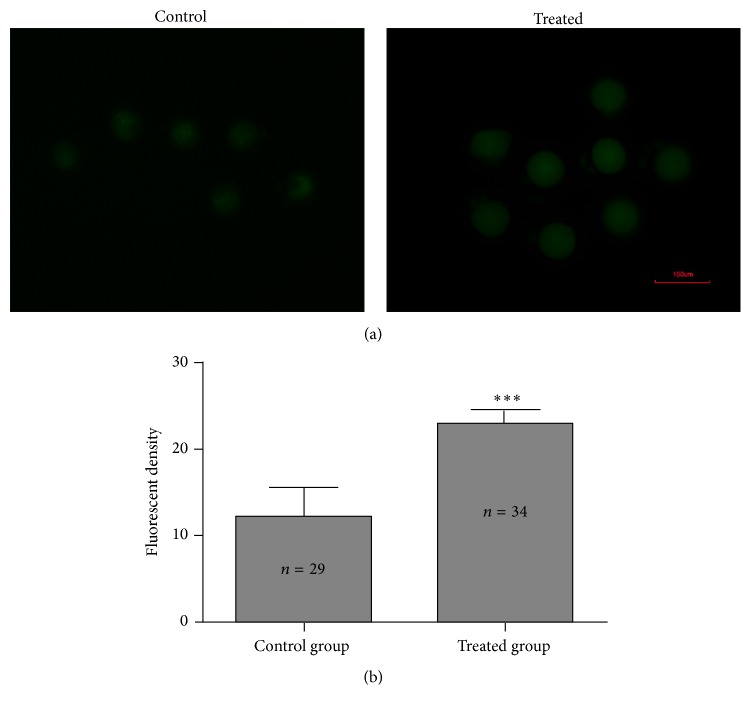
Comparisons of ROS levels between control group and H_2_O_2_ treated group. (a) Representative pictures of ROS levels. (b) Average fluorescence intensity per zygote. Data are showed as mean ± SD, collected from three independent experiments, and each experiment had 5 zygotes at least. *n* shows the number of zygotes. ^*∗∗∗*^ *P* < 0.001, the treated group compared with the untreated group (Student's *t*-test).

The comparison of the yields of ROS between treated and untreated group was via DCFH-DA fluorescence intensity in zygotes (7.5 hpi) (Figure 2(a)). The mean fluorescence intensity of ROS in zygotes of the treated group (23.01 ± 1.47) was about 2-fold higher than that in the control group (12.19 ± 3.44) (*P* < 0.05, Figure 2(b)).

#### 3.2.2. Change in MMP of Zygotes with Time

**Figure 3:**
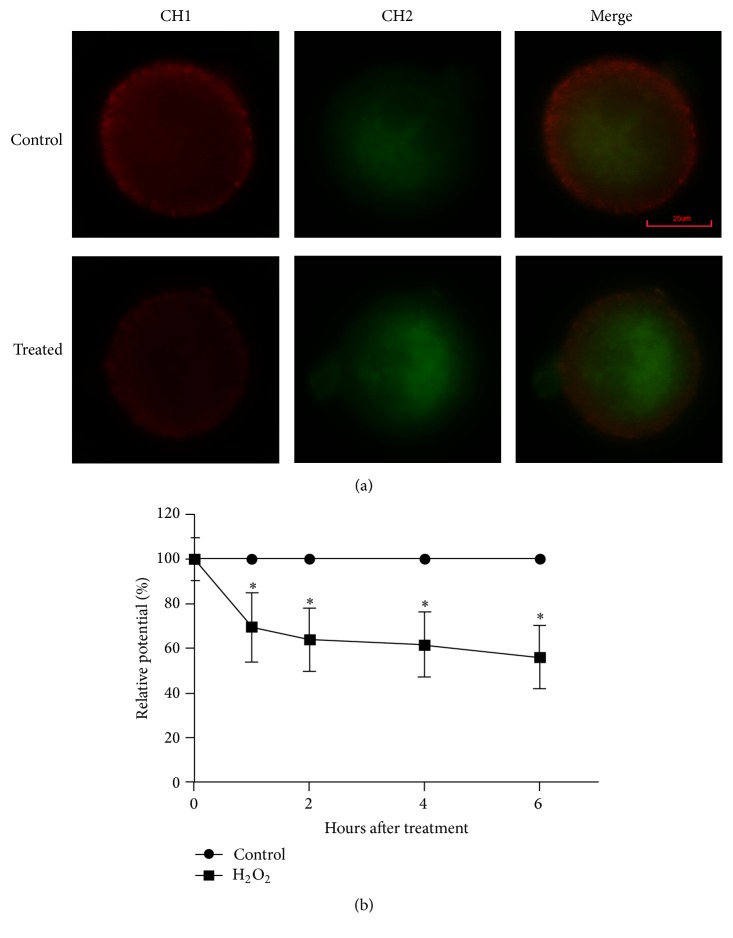
Dysfunction of mitochondria in mouse zygotes induced by H_2_O_2_. (a) Representative images of MMP in mouse zygotes at 6 h from treated group and control group; red fluorescence from channel 1 represented J-aggregates (high polarized mitochondria); green fluorescence from channel 2 represented monomer form of JC-1 (low polarized mitochondria). (b) The analysis of MMP was via the comparisons of relative fluorescence intensity and set the average value of red/green fluorescence intensity at each time point from control group as 100%; MMP in treated group was showed relative to control group at the corresponding point time. MMP in treated zygotes declined during the first hour after treatment by 0.03 mM H_2_O_2_ for 30 min and kept on dropping over the subsequent hours. Data are presented as mean ± SD in three independent experiments. ^*∗*^ *P* < 0.05, the treated group versus the control group (Student's *t*-test).

To further characterize the harmful effects of mild oxidative stress on embryo development in vitro, we detected the variation of MMP after treatment of zygotes with 0.03 mM H_2_O_2_ at 0, 1, 2, 4, and 6 h. The MMP was higher in control group than treated group at all check points (Figure 3(a)). The relative ratio of red to green fluorescence intensity indicated that MMP dropped rapidly within the first hour and did not recover within 6 hours after treatment (*P* < 0.05, Figure 3(b)). That is to say, the relative pixel ratio intensity, which reflected the MMP, was lower in H_2_O_2_-treated zygotes within 1 h after the treatment of H_2_O_2_ compared with the control group.

#### 3.2.3. Expression of *γ*H2AX in Embryos with Oxidative Stress Injury

**Figure 4:**
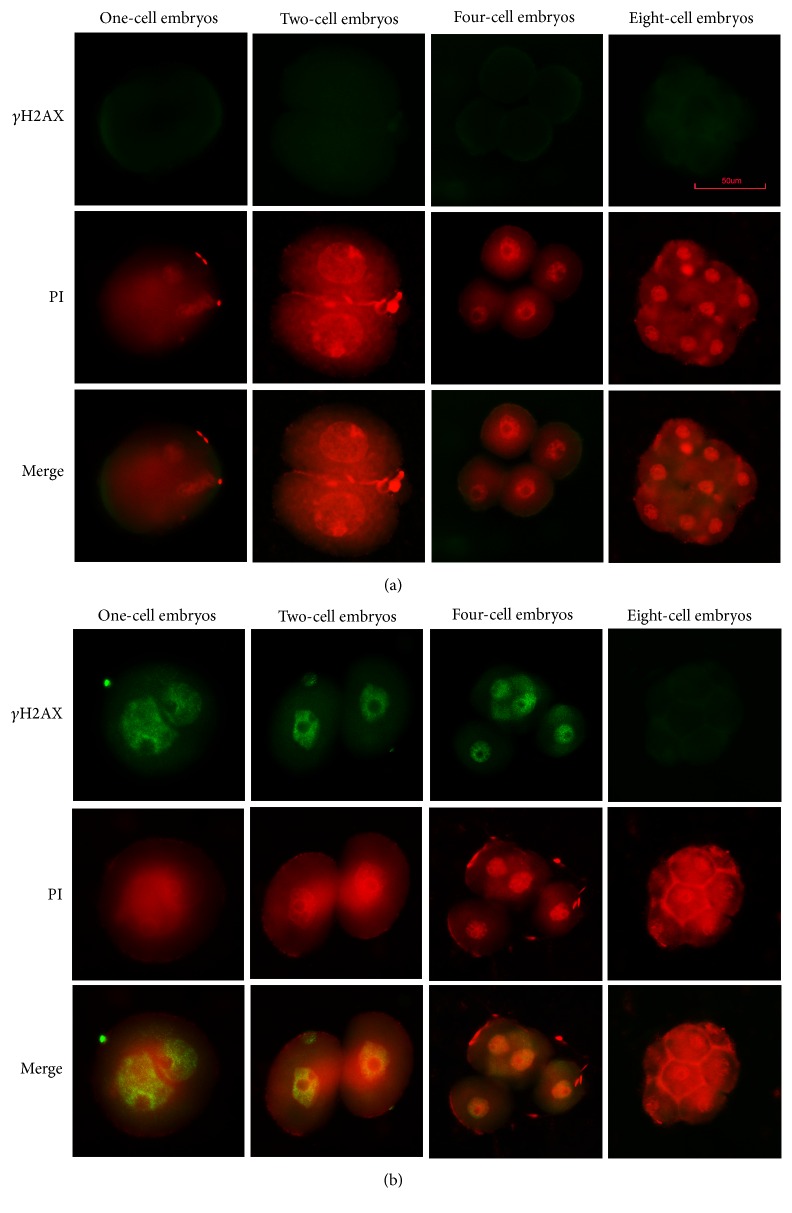
Expression of *γ*H2AX. (a) Control group; no positive signal. (b) 0.03 mM H_2_O_2_ treated group; positive signal at one-, two- and four-cell stages. PI = propidium iodide staining.

We discovered that H_2_O_2_-treated embryos expressed *γ*H2AX at the one-, two-, and four-cell embryos. However, there is no *γ*H2AX staining in control group (Figures 4(a) and 4(b)). Therefore, DNA damage existed in one-, two-, and four-cell embryos that developed from oxidative damaged zygotes. Representative images are shown in Figures 4(a) and 4(b).

#### 3.2.4. Apoptosis of Blastocysts

**Figure 5:**
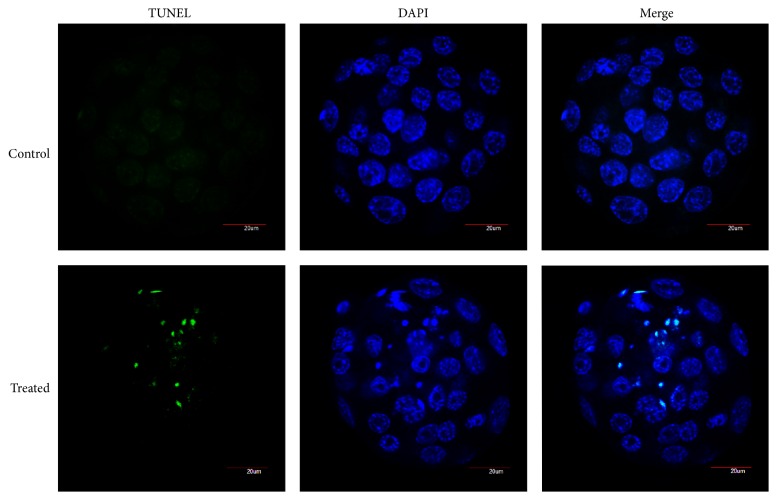
Hydrogen peroxide-induced apoptosis. Representative images of normal and apoptotic cells in mouse blastocysts; no positive signals (Green stains) appeared in control group; several positive signals could be seen in treated group. Nuclei were stained with DAPI (blue).

The average cell number per blastocyst in the treated group was 37.77 ± 3.94 and 46.74 ± 3.05 in the control group (*P* < 0.05). At the same time, the average apoptotic cell counts was over 2-fold higher in the treated group relative to the untreated group (6.24 ± 2.42 (treated) versus 2.27 ± 0.70 (control)) (*P* < 0.05). Moreover, the mean apoptotic rate was (15.9 ± 5.8)% for each H_2_O_2_-treated embryo versus (4.9 ± 1.3)% for the control. Representative images of normal and apoptotic mouse blastocysts are illustrated in Figure 5.

## 4. Discussion

Embryos cultured in vitro are subjected to oxidative stress. We used H_2_O_2_ to mimic oxidative stress. Finally, we gain a series of embryo models which suffer different levels of oxidative stress. And we found that a slightly high level of ROS could lead to a low formation rate of blastocyst with less cells by reducing MMP of zygotes and increasing DNA damage at one-, two-, and four-cell embryos.

Bain and colleagues reported that effects of H_2_O_2_ on blastocyst formation became more severe during the treatment of later stages of development [26]. Another important factor is embryos subjecting to oxidative stress from early stage in clinic. Thus, we chose zygotes rather than 2- or 4-cell embryos to be treated to avoid excessively adverse effects on embryos. Ciray et al. and Kasterstein et al. have shown that embryos in clinic (5% or 20% O_2_ concentration) have a similar cleavage rate at day 3 but decreasing blastocyst formation rate at day 5 or 6 in 20% O_2_ atmosphere [7, 8]. So we use small concentration gradient (0–0.1 mM) and low doses of H_2_O_2_ to treat zygotes for 30 minutes according to our preliminary experiment and other researches [10, 11, 13, 26], just for embryo model with similar developmental pattern above. Furthermore, cells undergoing G1→S transition are very sensitive to low levels of stimulus [22]. Many embryos under oxidative stress step into a transient cell cycle arrest which is activated by DNA damage response before apoptosis [11]. In order to gain a suitable model to research molecular mechanisms about DNA damage repair, cell cycle, and apoptosis in our subsequent experiments, we utilized H_2_O_2_ to treat zygotes at 7 hpi as they are in G1 phase [20], which is the first checkpoint of cell cycle to keep genetic stability. Also, that may provide models for us to study molecular mechanisms which activate G2/M phase delay.

Based on the theory above, we implemented experiment 1 and the results showed that zygotes presented no division in 0.1 mM H_2_O_2_ at 60 hpi. And there will be a lower percentage of embryo formation rate at each stage with treatment of a higher concentration of H_2_O_2_. To put it another way, embryo developmental patterns in 0.01–0.05 mM H_2_O_2_ could be used, as alternate models, to research influences of mild oxidative stress on developmental retardation. Developmental conditions of 0.01 or 0.02 mM H_2_O_2_ group could be used to study embryo development by Peng et al. No obvious differences were found in cleavage or high quality embryo rate at day 3 [27]. Moreover, the embryo model in 0.04 or 0.05 mM H_2_O_2_ group may suit studying the human embryo development with low formation rate at day 3 and day 5 or 6 [28]. Most importantly, we selected 0.03 mM H_2_O_2_ in our subsequent studies as we found the developmental style was similar to clinic and other researches [7, 8]. That is, there are no obvious differences in the percentage of cleavage rates between 20% and 5% O_2_ concentration, but 5% O_2_ group have more top-quality embryos on day 3 and more blastocysts on day 5 or 6 [7, 8]. Our data presented that embryo formation rates at 24 h and 48 h were approximate, but more blastocysts with less apoptotic cells were in control group than 0.03 mM H_2_O_2_. The concurrent research found G2/M delay of zygotes in 0.03 mM H_2_O_2_ group [29]. As is known human embryos at 4- to 8-cell and mouse embryos at 1- to 2-cell are undergoing ZGA (zygotic gene activation) phase, which is a key process of maternal to zygotic transition [29, 30]. Embryos develop the ability to support proper development of early stage during ZGA phase [30]. The above prompt is that potential damage may exist in early mouse embryos (1- and/or 2-cell embryos) in 0.03 mM H_2_O_2_ group and it may be adverse to the formation of blastocyst. Further investigations will be done to determine the molecular mechanisms about cell cycle arrest. As to reasons for the otherness of human embryo development aforementioned, it may be the inconsistent ovulation induction protocols, different lab conditions, technologist's experiences, technologies and skills, and many other uncontrollable factors. Additionally, we have observed that the shape of embryos in control group is larger than that of the treated group and that outcome may be induced by high apoptotic rate in blastocyst of treated group. Nevertheless, it needs more endeavor to unveil the influences of the above phenomenon on blastocyst quality. Our results showed a higher production of intracellular ROS (Figure 2(b)) which indicates that embryos are in oxidative stress status. Moreover, we could draw a conclusion from the sharp decline of MMP that zygotes were damaged during 1 h after treated by 0.03 mM H_2_O_2_. Embryos with continuous high MMP can reactive the arrest development [31]. Thus, embryos may be damaged by ROS at the first hour after treatment by 0.03 mM H_2_O_2_.

Our previous study revealed that development of zygotes from fresh spermatozoa and sperm treated with 1 mM H_2_O_2_ for 1 h was similar [19]. But blastocyst formation rate was significantly declined when treating zygotes with lower concentration of H_2_O_2_ and shorter processing time. It states that oxidative-stressed damage put more negative influences on zygotes than on sperm. And the reasons might be as follows: (1) two pronuclei were exposed to oxidative stress and both might be damaged; (2) embryonic cytoplasm was affected by ROS, especially mitochondria. Almost the whole cytoplasm is from oocyte and cytoplasm plays a major role in regulating early embryo development [10]. That finding further suggests that maternal resources are crucial in mediating early embryo development which conforms to other researches [29, 30].

To further delineate the effects of oxidative stress on DNA, we firstly detected the expression of *γ*H2AX. Our results showed that *γ*H2AX appeared at the one-, two-, and four-cell embryos in the treated group. Luo et al. found that mouse embryos exposed to 50-Hz sinusoidal electromagnetic fields (EMFs) for 24 h elicited the presence of *γ*H2AX foci in two-cell embryos or *γ*H2AX expressed in two- and eight-cell embryos with exposure for 48 h [32]. UV irradiation caused expression of *γ*H2AX at later embryo stage only [33]. Those behaviors of *γ*H2AX may be induced by various types of DNA damage or embryos diversely in response to different degree of DNA damage. Further investigations are necessary to better unveil that unsolved puzzle. G2/M checkpoint of zygotes was delayed at 0.03 mM H_2_O_2_ group as compared with control group [29]. And DNA damage exists in early embryos. There were no significant differences in the percentages of 2- and 4-cell embryo formation. One possible reason could be that G2/M phase is from 17 hpi to 20 hpi in control group [20], but we recorded the cleavage rate at 24 hpi, the last 4 hours before recording provide embryos of treated group with time to catch up. Another reason might be that oxidative stress, increasing the metabolism with superoxide detoxification, might accelerate the speed of the first cleavage [26]. Also DNA damage response may play a positive role in repair damaged DNA of early embryos, and it may benefit from the functions of *γ*H2AX below: firstly, *γ*H2AX may work as it collects other signal or repair proteins to injured sites [16, 17, 23]; secondly, it localizes at kinetochores and calls together MDC1, MDC2, and CDC20 for assembling an integrated mitotic checkpoint complexes [34]; finally, dynamic *γ*H2AX in cell cycle reminds us of its specific role in the process of mitosis [18, 35]. Recently, it has been demonstrated that PIk3-mediated activation of *γ*H2AX subsequently regulates the cell cycle progression and cell fate in human corneal epithelial cells [36]. Besides, *γ*H2AX accumulated beyond its role in DNA damage and as a regulator of cell cycle progression by inhibitor DNA replication [37, 38]. Those remind us that *γ*H2AX may involve in DNA damage response to regulate cell cycle in zygotes with oxidative-stress damage, too. We may learn that normal zygotes mediate G2/M by protein kinase A (PKA), WEE1B, 14-3-3*ε*, and M-phase promoting factors [39–41]. But the specific molecular pathways regulate cell cycle of normal zygotes and zygotes with oxidative-stressed damage are unclear, and whether DNA damage in zygotes activates cascade of ATM → Chk1 → Cdc25B/Cdc25C during G2/M to repair the damage depends on our further study. Moreover, compared with our previous study, in which *γ*H2AX appeared at one- and four-cell stage [19], the above phenomenon verifies that zygotes with oxidative-stressed damage exert more negative effects on embryo development than zygotes derived from ova fertilized with H_2_O_2_-treated sperm again. In the later stage that did not obviously express *γ*H2AX, we assume that damaged cells in those embryos were removed following either apoptosis or permanently developmental arrest or repairing their damaged DNA. Our results illustrated that ROS gives rise to the formation of blastocysts with less cells. Although DNA damage response may produce positive roles at one-, two-, and four-cell embryo stages, the cell counts of blastocyst and the blastocyst formation rate were still significantly lower than the untreated group, indicating that activation of DNA repair mechanisms and/or cell cycle arrest failed to completely repair the damage. Our results strongly state that adverse effects of ROS on embryo development, at least partly, act through DNA damage.

## 5. Conclusions

In brief, effects of oxidative stress on early embryos are dose-dependent and lasting to blastocyst. Also, the decline of MMP caused by oxidative stress in zygotes may forecast the compromised development of embryos. *γ*H2AX may play an important role in DNA repair of early embryo stages, but cells with unrepaired DNA will reduce the blastocyst formation rate. Therefore, our embryo models should be considered when evaluating the hazardous effects of environmental ROS in human embryos and search for effective approaches for prevention.
